# A Novel Approach to First-Rib Resection in Neurogenic Thoracic Outlet Syndrome

**DOI:** 10.3389/fsurg.2021.775403

**Published:** 2021-11-12

**Authors:** Yueying Li, Yanxi Liu, Zhan Zhang, Xuehai Gao, Shusen Cui

**Affiliations:** ^1^Department of Hand Surgery, China–Japan Union Hospital of Jilin University, Changchun, China; ^2^Department of Nursing, China–Japan Union Hospital of Jilin University, Changchun, China

**Keywords:** neurogenic thoracic outlet syndrome, piezo surgery, rib resection, complications, Disabilities of the Arm, Shoulder and Hand (DASH)

## Abstract

**Objectives:** The treatment for neurogenic thoracic outlet syndrome (NTOS) conventionally involves first-rib resection (FRR) surgery, which is quite challenging to perform, especially for novices, and is often associated with postoperative complications. Herein, we report a new segmental resection approach through piezo surgery that involves using a bone cutter, which can uniquely provide a soft tissue protective effect.

**Methods:** This retrospective study involved the examination of 26 NTOS patients who underwent piezo surgery and another group of 30 patients who underwent FRR using the conventional technique. In the patient group that underwent piezo surgery, the rib was first resected into two pieces using a piezoelectric device and subsequently removed. In the patient group that underwent conventional surgery, the first rib was removed as one piece using a rib cutter and rongeurs.

**Results:** The piezo surgery group had significantly shorter operative time (96.85 ± 14.66 vs. 143.33 ± 25.64 min, *P* < 0.001) and FRR duration (8.73 ± 2.11 vs. 22.23 ± 6.27 min, *P* < 0.001) than the conventional group. The posterior stump length of the residual rib was shorter in the piezo surgery group than in the conventional group (0.54 ± 0.19 vs. 0.65 ± 0.15 cm, *P* < 0.05). There were no significant differences in postoperative complications and scores of the Disabilities of the Arm, Shoulder and Hand (DASH) questionnaire, the Cervical Brachial Symptom Questionnaire (CBSQ), and the visual analog scale (VAS). Even the TOS index (NTOS Index = [DASH + (0.83 × CBSQ) + (10 × VAS)]/3) and patient self-assessments of both the groups showed no significant differences. Univariate analyses indicated that the type of treatment affected operative time.

**Conclusion:** Our results suggest that piezo surgery is safe, effective, and simple for segmental FRR in NTOS patients. Piezo surgery provides a more thorough FRR without damaging adjacent soft tissues in a relatively short duration and achieves similar functional recovery as conventional techniques. Therefore, piezo surgery can be a promising alternative for FRR during the surgical treatment of NTOS.

## Introduction

Neurogenic thoracic outlet syndrome (NTOS) is a compressive neuropathy caused by brachial plexus compression in the thoracic outlet region (1–3). The first rib can often cause the compression of the brachial plexus (4, 5), and its removal is considered effective for thoracic outlet decompression (6).

Previous studies defined first-rib resection (FRR) as the most demanding and potentially dangerous component of TOS surgery (6, 7). A close relationship between the first rib and soft tissue can be associated with soft tissue injury, including pneumothorax (with an incidence of 2.5–10%), lymph leakage (incidence of 2–9.3%), and nerve injury (incidence of 11%) (8–18). Therefore, several methods for the minimal invasive resection of the first rib have been reported including video assisted thoracic surgery (VATS), and robotic-assisted thoracic surgery (19, 20). Zehnder et al. (21) and colleagues have reported a transthoracic video-assisted robotic approach with modified 3-port for first rib resection, brings minimally invasive mode with the fewest number of incisions. In addition, rib resection with a sufficient length is vital for NTOS decompression. The rib should be severed as posteriorly as possible, preferably at the junction with the transverse process, and as anteriorly as possible, ideally at the costochondral junction (3, 22). Removing the first rib in one piece within this range is a unique challenge in decompression procedures for NTOS because during vascular thoracic outlet syndrome decompression, only resection of the anterior portion is required (23, 24). Moreover, the posterior part of the first rib is rather deep, and the brachial plexus should be significantly retracted for adequate exposure of this portion, which can cause intraoperative nerve traction injury. Current instruments used for FRR include the Schumacher bone cutter, the Raney Rongeur, and Kerrison and duckbill rongeurs. These are operated manually and require a large working space; this affects the operational field and increases the risk of soft tissue injury, making the conventional FRR technique more challenging, especially for novices (3, 22, 23). To overcome these issues, we developed a sectional removal technique using an ultrasonic bone cutter, i.e., piezo surgery. Piezo surgery is an innovative technique that implements a specific frequency to cut bone, rather than soft tissue, to avoid damaging the surrounding soft tissues (25, 26). The piezo surgery device has a small operating head that takes up less working space. The improved visualization negates the need for forceful nerve retraction (27–31). We resected the first rib via three cuts. The removal of two smaller pieces is much easier than that of one lengthy piece. Piezo surgery is simple and straightforward even for novices (26, 32).

In this study, we examined NTOS patients who underwent FRR either through this new method or through the conventional technique. We compared the operative time, FRR duration, length of the posterior rib stump, length of hospitalization, complications, and results of physical examinations and scores on functional questionnaires between the two groups. Aim to provide a viable alternative for FRR in terms of safety and convenience.

## Materials and Methods

### Preoperative Evaluation and Patient Selection

Following ethics approval from the Institutional Review Board of the China–Japan Union Hospital of Jilin University (code 2021-KYLL-060020, date June 2, 2021), we performed a retrospective chart review of patients with NTOS who had undergone FRR between March 1, 2010 and March 1, 2018. The study enrolled 56 patients (59 operations). The requirement for patient consent was waived due to the retrospective nature of the study. This research was conducted in accordance with the principles of the Declaration of Helsinki and its later amendments.

For all patients, surgery (conventional or piezo surgery) was performed after conservative management failed for at least 3 months. Cervical fluoroscopy and computed tomography were performed before surgery. Patients with a wide and vertical first rib were chosen to perform the FRR (33). All patients underwent FRR using a modified supraclavicular approach (34). Patients with a diagnosis of vascular TOS, cervical rib, distal nerve entrapment, cervical disc diseases, frozen shoulder, or neurogenic pectoralis minor syndrome were excluded from this study. Patients with symptoms suggestive of central nervous system diseases that might confound NTOS symptoms were also excluded. Patients who missed follow-up assessments or did not follow the postoperative physical therapy (PT) protocol were also excluded. The detailed descriptions of the NTOS diagnostic criteria implemented in our study are similar to those of previously published studies (34).

Patients were divided into two groups: the piezo surgery group, in which FRR was segmentally resected with piezo surgery, and the conventional instrument group, in which FRR was resected as one piece with a conventional rib cutter and rongeurs. Between March 1, 2010 and December 31, 2014, conventional instruments were used; the piezo surgery technique was introduced and applied from January 1, 2015 to March 1, 2018.

### Surgical Technique

General endotracheal anesthesia was induced with the patient in the supine position, following which the patient's neck was extended and turned to the opposite side. A modified supraclavicular incision extending to the deltopectoral groove was made, as previously reported (34). The scalene fat pad was mobilized to expose the anterior scalene muscle, phrenic nerve, brachial plexus nerve roots, and middle scalene muscle. The lateral aspect of the first rib was palpated and visualized, and the long thoracic nerve was identified.

**Figure 1:**
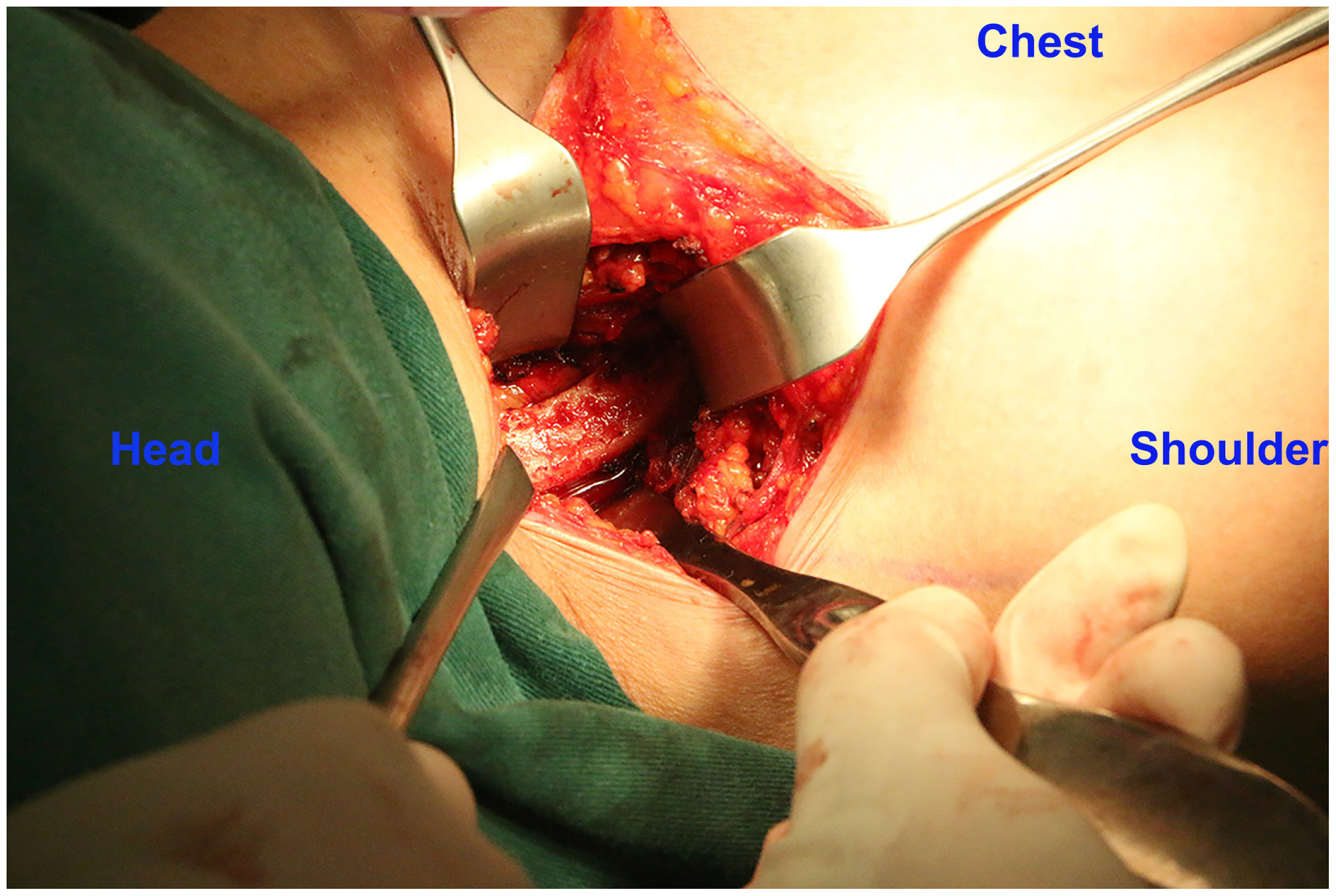
Image of the partial exposure of the first rib. The brachial plexus is retracted medially, and the middle scalene muscle is partially excised and detached to expose the first rib.

**Figure 2:**
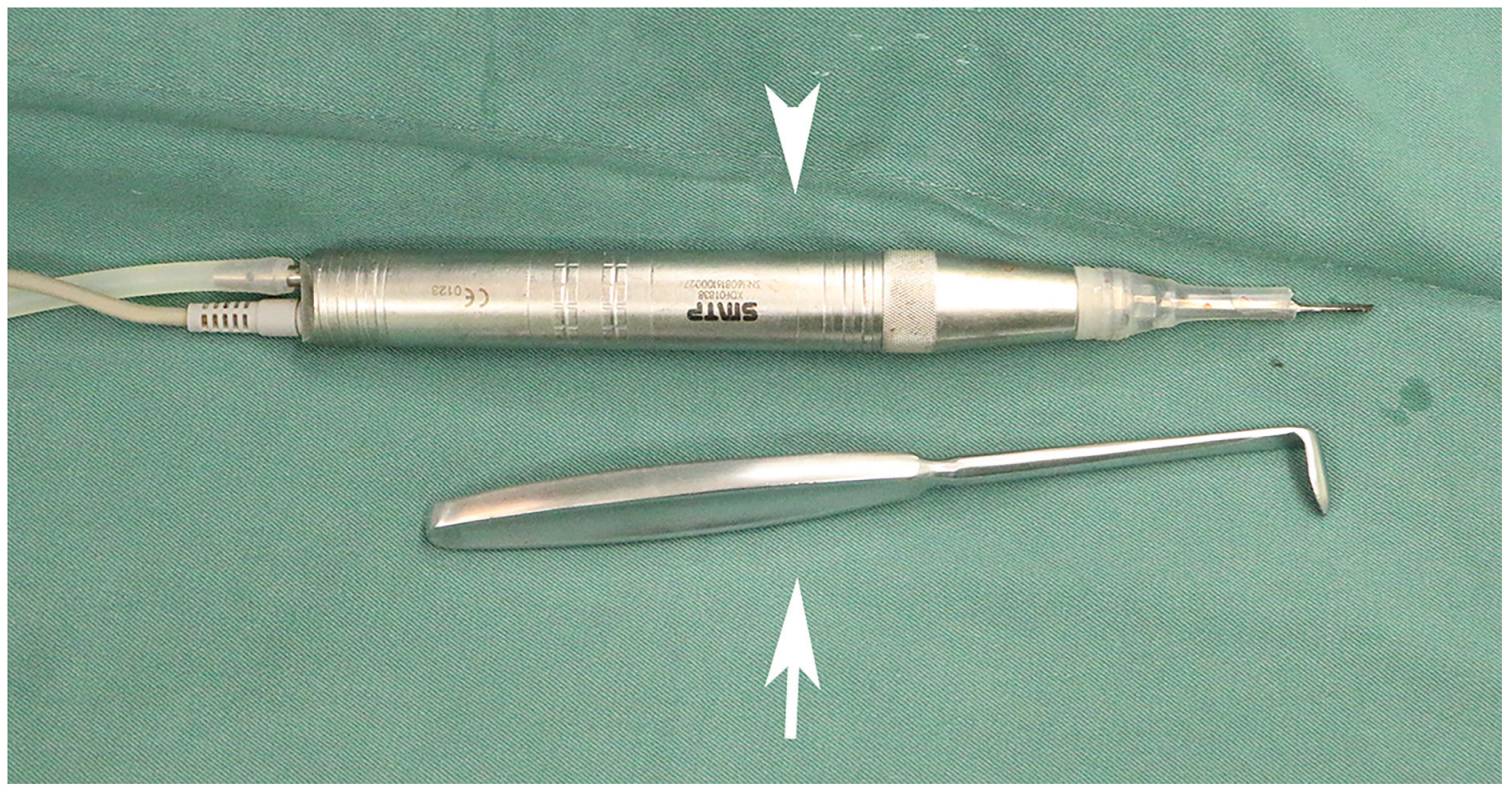
Image of the piezoelectric device and modified periosteal elevator used during the removal of the first rib. The piezo surgery medical device with a slender head (represented by an arrowhead) is shown here. The modified periosteal elevator (represented by a white arrow) was used for blunt dissections. This provides a protective edge to minimize injuries to neurovascular structures surrounding the first rib.

**Figure 3:**
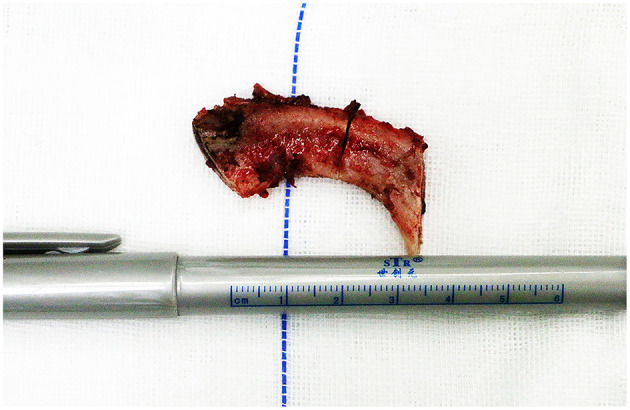
An image of removed rib sections. The first rib was transected into two pieces for easier removal. The recombinant first rib segments are shown here.

The anterior scalene muscle was divided and removed, the brachial plexus was separated, and complete external neurolysis was performed by removing all fibroinflammatory scar tissue around the nerve roots. With the brachial plexus retracted medially, the middle scalene muscle was partially excised and detached from the posterior surface of the first rib (Figure 1). The first rib evaluation was done with respect to the feature and the direction. Furthermore, the relationship between the first rib and brachial plexus was assessed statically and dynamically on movement of the upper extremity; if the nerve was in contact with the rib, the rib was removed (35). Finally, the wide first rib in vertical direction along with brachial plexus compression were indicated to be removed. A modified periosteal elevator (Figure 2) was used to separate the extra-pleural fascia and attachments of the intercostal muscles around the undersurface of the first rib. This provided a protective edge to help minimize the risk of injury to the neurovascular structures and pleura surrounding the first rib. In the conventional instrument group, the first rib was transected anteriorly near the costochondral junction and posteriorly close to the transverse process in one piece with a rib cutter and rongeurs. In the piezo surgery group, the first rib was transected anteriorly near the costochondral junction medial to the scalene tubercle and then transected in the middle, after which a posterior section was made as close to the transverse process as possible using the piezo surgery medical device (SMTP Technology®, Beijing, China) (Figure 2). The intercostal muscles along the posterolateral aspect of the first rib were divided using electrocautery, and the ribs were removed in sections (Figure 3; Supplementary Video). The rongeurs were occasionally used to trim stumps. Removing the first rib allowed the neurovascular bundle to descend into the pleural space; all its components were then identified. Hemostasis was performed, a suction drain was left in place, and layered closure was performed.

The same postoperative PT protocol was followed for each patient. Patients with poor compliance with PT, which was defined as completing less than 50% of the total required amount of PT (fewer than 3 days a week or less than one whole set per day) were excluded from the analysis, as previously described (34).

### Outcome Measurements

Postoperative cervical and chest fluoroscopy and computed tomography were performed to evaluate the excision of the first rib, as well as diaphragmatic function.

Postoperative outcomes were assessed with respect to operative time, FRR duration, length of the posterior rib stump, length of hospital stay, complications, the Disabilities of the Arm, Shoulder and Hand (DASH) questionnaire, the Cervical Brachial Symptom Questionnaire (CBSQ), the visual analog scale (VAS), TOS index (NTOS Index = [DASH + (0.83 × CBSQ) + (10 × VAS)]/3) and each patient's self-assessment (reported as resolved, markedly improved, fair, or poor, depending on the degree of overall recovery; detailed criteria are presented in the Supplementary Material (Supplementary Table 1).

### Statistical Analysis

The Shapiro–Wilk test was initially implemented to confirm the normal distribution of our data. A paired t-test was used to compare differences in group means for paired samples. Normally distributed data are expressed as means ± standard deviations and were compared using independent t-tests. Non-normally distributed data are presented as medians and ranges and compared using the Mann–Whitney U test. Categorical variables were compared using the chi-square test and Fisher's exact test. Univariate and multivariate analyses were conducted to identify factors independently associated with scale scores. The following parameters were examined: age, sex, trauma history before symptom onset, duration of the presence of symptoms prior to surgery, physical examinations, and functional scores. Statistical significance was set at *P* < 0.05 for all tests. Statistically significant (*P* < 0.05) or nearly statistically significant (*P* < 0.10) variables in the univariate analysis were entered into a multivariate analysis using stepwise multiple regression.

## Results

### Study Population and Pretreatment Characteristics

**Figure 4:**
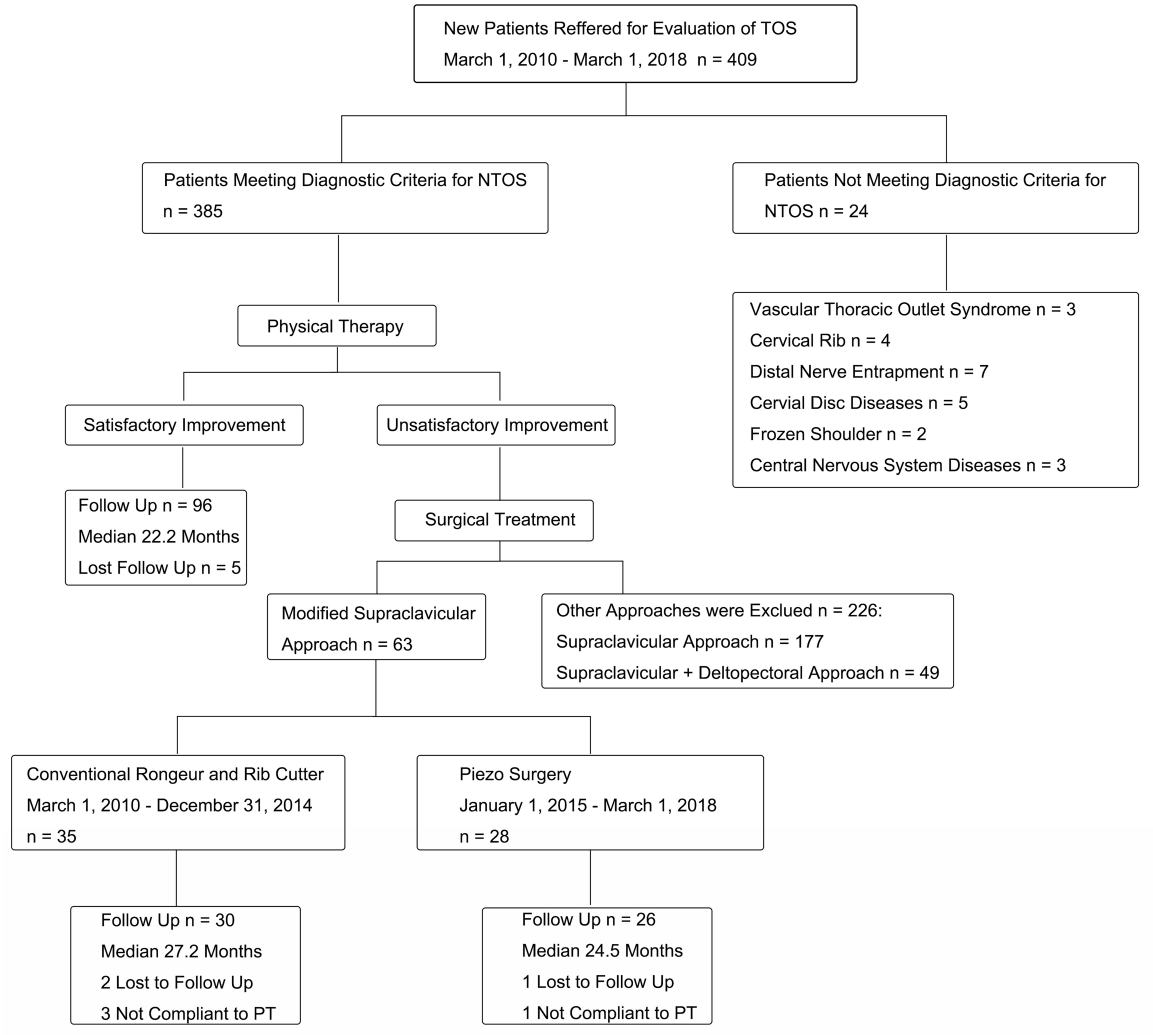
The flow diagram of patient selection of the study. TOS, thoracic outlet syndrome; NTOS, neurogenic thoracic outlet syndrome.

Among the initial cohort of 385 patients meeting the diagnostic criteria for NTOS, 96 (25%) obtained satisfactory symptom improvement with the initial PT trial and chose to continue conservative management. A total of 289 patients (75%) experienced insufficient improvement with PT and elected to undergo surgery. Among the 289 surgical cases, 63 included a modified supraclavicular approach; cases involving other surgical approaches were excluded from the study. Of the 63 patients, 35 underwent the conventional surgery (forming the conventional group) and the remaining 28 patients underwent surgery via the novel method (forming the piezo surgery group). Two patients from the conventional group and one from the piezo surgery group missed follow-up assessments; hence, they were excluded from the analysis. Further, three more patients from the conventional group and one patient from the piezo surgery group were excluded owing to discrepancies in following the PT protocol. Ultimately, 30 patients (31 surgeries including one bilateral) from the conventional group and 26 patients (28 surgeries including two bilateral) from the piezo surgery group were included in this analysis (Figure 4).

There were no significant differences in the presenting characteristics between the conventional and piezo surgery groups in terms of age, sex, previous injury, symptom duration, physical examination, pretreatment DASH scores, CBSQ scores, VAS scores, TOS index, hospital stay, or postoperative complications (Table 1).

**Table 1:** Patient characteristics at presentation in the conventional group and piezo surgery group.

**Characteristic**	**Conventional group (30)**	**Piezo surgery group (26)**	***P*** **value**
Age, years	38.6 ± 13.14	40.38 ± 10.89	0.586
Female gender	23	20	0.982
Neck trauma before onset			0.712
Yes	13	10	
No	17	16	
Bilateral NTOS	1	2	0.470
Median duration prior to op in months (range)	28.37 (3–250)	15.08 (3–172)	0.132
Examination			
Scalene tenderness	22	20	0.155
Positive EAST	25	23	0.584
Positive ULTT	21	19	0.799
Positive hyperabduction maneuver	29	22	0.115
Positive costoclavicular maneuver	27	25	0.373
Abnormal ulnar sensation of forearm	28	23	0.524
Weakness of handgrip strength	20	21	0.235
Weakness of interossei strength	25	24	0.311
Scores before op			
DASH	52.26 ± 21.18	54.25 ± 20.92	0.725
CBSQ	66.63 ± 27.99	71.69 ± 29.82	0.518
VAS	5.7 ± 3.2	5.32 ± 3.13	0.653
TOS index	54.87 ± 21	55.65 ± 14.22	0.873
Abnormal Ulnar sensory neural action potential	7	6	0.617
Abnormal latency of the ulnar F-wave/ Ulnar F response	18	14	0.423
Abnormal medial antebrachial cutaneous nerve response	9	11	0.249
Operative time (min)	143.33 ± 25.64	96.85 ± 14.66	**0.000**
First rib resection duration (min)	22.23 ± 6.27	8.73 ± 2.11	**0.000**
Posterior stump length of first rib (cm)	0.65 ± 0.15	0.54 ± 0.19	**0.024**
Hospital stays (days)	2.43 ± 1.19	2.12 ± 0.95	0.281
Technique-related complications	2	0	0.18
Lymph leakage	1	0	
Hemorrhage	1	0	
Median FU in months (range)	27.37 (12–67)	21.77 (12–60)	0.116

*NTOS, neurogenic thoracic outlet syndrome; EAST, elevated arm stress test; ULTT, technique-related complications; DASH, Disabilities of the Arm, Shoulder and Hand; CBSQ, Cervical Brachial Symptom Questionnaire; VAS, visual analog scale; TOS, thoracic outlet syndrome; FU*.

### Piezo Surgery and Operation Efficacy

**Figure 5:**
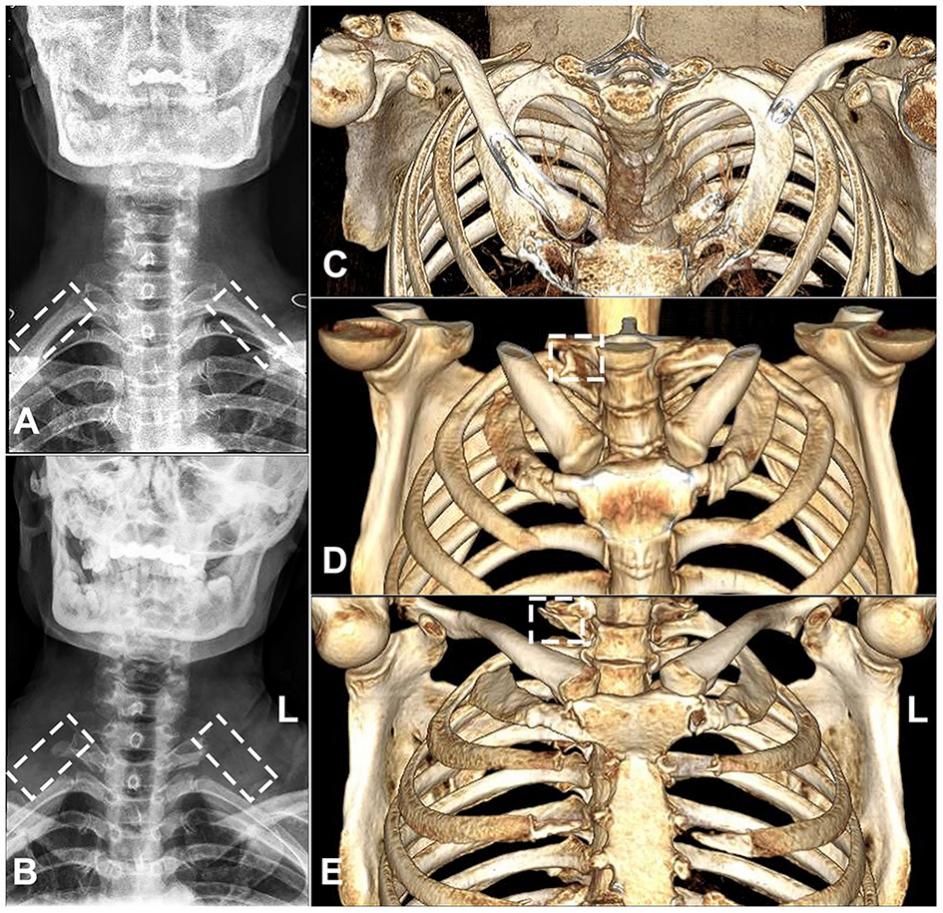
Images of cervical fluoroscopy and a computed tomography scan of the first rib. **(A)** A representative image of preoperative cervical fluoroscopy of the involved first rib at the T1 level. **(B)** Postoperative cervical fluoroscopy image showing the bilateral removal of the proximal part of the first ribs (nearly up to their articulation) with transverse processes (represented by dashed rectangles). **(C)** A flat panel computed tomography 3D image showing a preoperative image of the first rib. **(D)** The posterior stump of the first rib (with an irregular surface) in the conventional surgery group (represented by a dashed square). **(E)** The posterior stump of the first rib (with a smooth surface) in the piezo surgery group (represented by a dashed square).

We compared the differences between the two groups in mean operative time, FRR duration, and posterior stump length of the first rib. The mean operative time of the piezo surgery group was 96.85 ± 14.66 min, whereas that of the conventional group was 143.33 ± 25.64 min (*P* < 0.001). The FRR duration of the piezo surgery group was 8.73 ± 2.11 min, whereas that of the conventional group was 22.23 ± 6.27 min (*P* < 0.001). The posterior stump length of the first rib was shorter in the piezo surgery group (0.54 ± 0.19 cm) than that in the conventional group (0.65 ± 0.15 cm; *P* = 0.024) (Table 1; Figure 5). Univariate analyses demonstrated significant correlations between the operative time and the type of treatment (r = 0.818; *P* < 0.001).

### Follow-Up and Treatment Outcomes

The mean postoperative follow-up period was 27.2 (12–67) months for the conventional group and 24.5 (12–60) months for the piezo surgery group. Although significant improvement was seen in both groups, postoperative DASH scores, CBSQ scores, VAS scores, TOS indices, and patient self-assessments were not significantly different between the two groups. Two patients of the conventional group presented with complications (one with lymph leakage and the other with hemorrhage), both of which were resolved through reoperation. No patients in the piezo surgery group developed technique-related complications (Table 2).

**Table 2:** Functional outcomes of the conventional group and piezo surgery group.

**Assessment**	**Conventional group (30)**	**Piezo surgery group (26)**	***P*** **value**
Quantitative			
DASH	20.66 ± 16.39	22.65 ± 14.80	0.638
CBSQ	29.87 ± 25.25	25.65 ± 22.01	0.512
VAS	2.49 ± 2.71	1.81 ± 1.87	0.290
TOS index	23.30 ± 20.37	18.67 ± 9.42	0.292
Abnormal ulnar sensation of forearm	4	3	0.582
Weakness of handgrip strength	5	3	0.438
Weakness of interossei strength	8	3	0.139
Qualitative			0.495
Resolved	13	15	
Significant improvement	9	8	
Fair	4	2	
Poor	4	1	

*DASH, Disabilities of the Arm, Shoulder and Hand; CBSQ, Cervical Brachial Symptom Questionnaire; VAS, visual analog scale; TOS, thoracic outlet syndrome*.

## Discussion

In this study, we compared the segmental resection of the first rib through piezo surgery and one-piece resection using a conventional instrument. Our results indicated that piezo surgery is a safe alternative for FRR, with a shorter operative time and sufficient length resection. Patients in the two groups demonstrated similar surgical outcomes, as reflected by functional outcomes and patients' self-assessments; none of the patients in the piezo surgery group presented with technique-related postoperative complications; the operative time and FRR duration were significantly less (8.73 ± 2.11 vs. 22.23 ± 6.27) in the piezo surgery group than in the conventional group. Postoperative radiological examination demonstrated that the posterior stump length of the first rib in the piezo surgery group was shorter than that in the conventional group.

Same as the traditional supraclavicular approach, our modified supraclavicular approach has a relatively good access to the brachial plexus (21). Meanwhile, this modified approach can expose both supra- and infraclavicular areas, allowing us to visualize the subclavius muscle and resect it if it also compresses the brachial plexus in the infraclavicular region (34). However, even with this modified approach, safe exposure of the entire rib is still limited. Besides, intense traction of the neurovascular bundles is often required for adequate exposure in operation. With the help of piezo surgery, first rib can be managed easily in a safe mode with sufficient length.

The most outstanding advantage of piezo surgery is its soft tissue protective effect. Owing to the close relationship between the first rib and soft tissues (8), traditional FRR via the supraclavicular approach is usually associated with soft tissue injury, including pneumothorax (9, 10), neurovascular bundle injury (18, 36), sympathetic chain injury (9), and lymph leakage (11–16). In our study, there were no reports of soft tissue injury in the piezo surgery group. However, in the conventional group, one case of lymph fluid leakage and another of hemorrhage were reported, which required resolution by reoperation. In the patient with lymph fluid leakage, we found a small hole in the branch of the lymphatic vessel and repaired the damaged lymphatic vessel through microsurgery; for the patient with hemorrhage, we found blood oozing from the irregular posterior stump of the first rib and smoothened the fixation of the stump using rongeurs and smeared bone wax on the stump.

Since piezo surgery was first developed in 1988 by Italian oral surgeon Tomaso Vercelloti, it has been widely used in oral and maxillofacial surgery and otolaryngology-head and neck surgery as reported (37–39); moreover, to the authors' knowledge, the piezo surgery was not used previously in FRR. The piezoelectric ultrasound provides bone cutter ultrasonic micro-vibrations of 60–210 μm at 25–30 kHz, which allows for minimally invasive resection of the first rib without damaging soft tissues (28, 29). In addition, the stump of the first rib is relatively smooth, which facilitates the efficacy of smearing bone wax following piezo surgery to prevent bleeding.

Piezo surgery is a simple and user-friendly technique. Previous publications reported that the first rib is usually removed in one piece (6, 7, 23). The bottom of the first rib closely adheres to the pleura and requires long-distance detachment to be performed without direct vision. Segmental resection of the first rib is recommended to remove the first rib easily; however, this is difficult when using conventional instruments (3, 24, 34, 40). With the help of piezo surgery, the rib can be cut thrice into two pieces more easily, clearly, and without generating heat due to the cavitation effect followed by the divisions of the rib. This creates a trap-door configuration at two sites, thereby enlarging the operation field (26, 32, 41, 42). Additionally, the piezo surgery device has a slender head, which can be used in narrow and deep spaces with high dexterity and provides easier access to cut the first rib as close to the costotransverse joint as possible. These advantages reduce the operative time effectively, especially for patients with a wide first rib (43–46).

Because of the high dexterity of the piezo device, the surrounding tissue needs no excessive retraction during FRR. Various reports stated that remnant long posterior stumps of the first rib often led to NTOS recurrence (47–51). Moreover, FRR should be performed as posteriorly as possible (17, 18, 24, 47, 49). In our study, the posterior first-rib stump of patients in the piezo surgery group was dissected as closely as possible to its articulation with the transverse process and without intense nerve retraction, and the average length of the residual stump was shorter than that in the conventional group.

Our results showed that piezo surgery has many prominent advantages, including soft tissue protection, user-friendliness, and high dexterity. Nevertheless, our study also had some limitations. First, this was a nonrandomized retrospective study; thus, our ability to draw causal inferences is limited. Likewise, we were limited by the timeline of the implementation of different methods at our medical center, as piezo surgery was introduced at our institution much later than the conventional technique. All surgeries were done by the same surgeon, which may cause the bias of operative length. To exclude the bias caused by surgical experience, we choose the learning curve plateau period of the surgeon. Furthermore, we compared the FRR duration to exclude the impact of other procedures in the whole surgery. Future studies should aim to compare the conventional method and piezo surgery during the same period as this would help in inherently adjusting for time-dependent potential confounders. An additional limitation is that both groups had a small sample size. A unified operation approach may be necessary to compare the operation time effectively between the two groups.

In conclusion, our results show that piezo surgery is a safe, less damaging to soft tissue, effective, and easy-to-perform technique for FRR in patients with NTOS. Piezo requires a shorter operation duration, provides more thorough FRR without damaging adjacent soft tissues. Therefore, piezo surgery can be a promising alternative for FRR during the surgical treatment of NTOS.

## Data Availability Statement

The original contributions presented in the study are included in the article/Supplementary Material, further inquiries can be directed to the corresponding author/s.

## Author Contributions

SC: conceptualization, supervision, funding acquisition, and writing—review and editing. ZZ and XG: methodology. YLi: software and writing—original draft preparation. SC, YLi, YLiu, XG, and ZZ: validation. YLi and YLiu: formal analysis and project administration. ZZ and SC: investigation. XG, ZZ, and SC: resources. YLiu: data curation. ZZ: visualization. All authors have read and agreed to the published version of the manuscript.

## Funding

This research was funded by the Jilin Scientific and Technological Development Program (Grant No. 20160101077JC to SC), the Jilin Scientific and Technological Development Program (Grant No. 20190905003SF to SC), the Jilin Provincial School Joint Construction Special Project (Grant No. SXGJQY2017-13 to SC), the National Natural Science Foundation of China (Grant No. 81671220 to SC), the Industrial Independent Innovation Capability Project of Jilin Development and Reform Commission (Grant No. 2019C005 to SC), and the National Key Research and Development Program of China (Grant No. 2016YFC1101602 to SC). The funding sources played no role in study conception and design, data analysis or interpretation, paper writing, or deciding to submit this paper for publication.

## Conflict of Interest

The authors declare that the research was conducted in the absence of any commercial or financial relationships that could be construed as a potential conflict of interest.

## Publisher's Note

All claims expressed in this article are solely those of the authors and do not necessarily represent those of their affiliated organizations, or those of the publisher, the editors and the reviewers. Any product that may be evaluated in this article, or claim that may be made by its manufacturer, is not guaranteed or endorsed by the publisher.
