# Supplementary material for: A Novel Approach to First-Rib Resection in Neurogenic Thoracic Outlet Syndrome
Source: Front Surg. 2021 Nov 12;8:775403. doi: 10.3389/fsurg.2021.775403 (PMC8632710; doi:10.3389/fsurg.2021.775403)
Supplement: Supplementary file 1 [file Data_Sheet_1.DOCX]

**Table S1:** Criteria of self-assessment

| Status | Neurogenic Symptoms | Daily Activity | Work Performance |
| --- | --- | --- | --- |
| Resolved | Totally disappeared | Have no difficulty | Not affect |
| Markedly improved | Mostly disappeared, but the patient still expressed minor complaints | Have no difficulty | May or may not affect |
| Fair | Many symptoms remained | Affect | Affect |
| Poor | No relief was achieved or recurrent | Severely affect | Severely affect |

# Video caption: First rib resection with piezo surgery.
